# Effects of astaxanthin on microRNA expression in a rat cardiomyocyte anoxia-reoxygenation model

**DOI:** 10.3389/fphar.2023.1103971

**Published:** 2023-02-03

**Authors:** Xinxin Zhang, Min Xu, Shuilin Cai, Bei Chen, Hetong Lin, Zhiyu Liu

**Affiliations:** ^1^ College of Food Science, Fujian Agriculture and Forestry University, Fuzhou, China; ^2^ Key Laboratory of Cultivation and High-Value Utilization of Marine Organisms in Fujian Province, Fisheries Research Institute of Fujian, National Research and Development Center for Marine Fish Processing, Xiamen, China; ^3^ Engineering Research Centre of Fujian-Taiwan Special Marine Food Processing and Nutrition, Ministry of Education, Fuzhou, China; ^4^ College of Ocean Food and Biological Engineering, Jimei University, Xiamen, China

**Keywords:** astaxanthin, ischemia-reperfusion injury, microRNA, target genes, antioxidant activity

## Abstract

**Introduction:** The protective effects of astaxanthin against myocardial ischemia-reperfusion injuries are well documented, although the mechanisms are not defined.

**Methods:** The anoxia-reoxygenation injury model was established after astaxanthin treated H9c2 cells for 24 h. Cell viability, lactate dehydrogenase, oxidative stress level and western blot were tested. Secondly, measured the effects of astaxanthin pretreatment on microRNA expression in a rat myocardial cell anoxia-reoxygenation injury model.

**Results:** After anoxia-reoxygenation injury, in a dose dependent manner, astaxanthin increased cell viability, superoxide dismutase and glutathione peroxidase activity, decreased lactate dehydrogenase and malondialdehyde levels, downregulated protein expression of caspase-3, caspase-8, nuclear factor erythroid-2-related factor 2 and heme oxygenase-1, and upregulated the Bcl-2/Bax ratio. High-throughput sequencing and qPCR showed that microRNAs rno-miR-125b-5p and rno-let-7c-1-3p were differentially expressed (|log2| ≥ 0.585, q < 0.1) between the normal, anoxia-reoxygenation, and astaxanthin (1.25 μM) groups. Kyoto Encyclopedia of Genes and Genomes and GO Gene ontology pathway enrichment analyses showed that TNF signaling, axon guidance, NF-κB signaling pathway, and other pathways displayed differentially expressed microRNA target genes associated with myocardial injuries.

**Discussion:** These results suggested that thetarget genes of rno-miR-125b-5p were enriched in inflammation and apoptosis-related signaling pathways. Also, the results imply that simultaneous targeting of these related signaling pathways could significantly prevent myocardial anoxia-reoxygenation injury in the presence of astaxanthin.

## 1 Introduction

Astaxanthin, 3,3′-dihydroxy-β,β-carotene-4,4′-dione, occurs in shrimp and crab shells, red hair fruit yeast, rainbow-sounding red algae, fish, and the showy feathers of birds (Fassett and Coombes, 2011). Astaxanthin is composed of a six-node ring structure of four isoprene units linked by a conjugated double bond with one isoprene unit at each end. The conjugated polyene structure of the astaxanthin molecule is susceptible to oxidation and extremely unstable; thus, astaxanthin possesses high antioxidant activity as a quencher of singlet oxygen. Astaxanthin is reported to have anti-aging, anti-cancer, immune system regulation, and cardiovascular disease prevention properties (Zhang and Wang, 2015; Eren et al., 2019; Sztretye et al., 2019; Yin et al., 2021). Notably, Chen et al. (2020) found that astaxanthin had significant protective and damage mitigation effects in prevention and treatment of cardiovascular disease.

Myocardial ischemia-reperfusion (I/R) injury is a pathological process in which the myocardium is damaged after partial or complete acute blockage of the coronary arteries followed by restoration of normal perfusion (Wei et al., 2014). The mechanism of I/R injury appears to be related to a massive production of intracellular oxygen free radicals, calcium ion overload, and the inflammatory effect of leukocytes. Several basic studies have validated the ability of astaxanthin pretreatment to alleviate oxidative stress, apoptosis, and inflammatory responses induced by myocardial anoxia-reoxygenation (A/R) injury (Fassett and Coombes, 2009; Gai et al., 2020; Kohandel et al., 2021).

MicroRNAs are small, endogenous, non-coding RNAs composed of 19–25 nucleotides. MicroRNAs post-transcriptionally regulate gene expression, and their regulatory networks are involved in many biological processes (Chen et al., 2019), such as embryogenesis (Wójcik et al., 2017), cell proliferation and differentiation (Miao et al., 2017; Wu et al., 2018), apoptosis (Koo and Kwon, 2018), and tumorigenesis (Honardoost and Rad, 2018). In the cardiovascular system, microRNAs regulate growth and contraction of cardiomyocytes (Alikunju et al., 2022), cardiac rhythm (Briasoulis et al., 2019; Cao et al., 2019), plaque formation (Meng et al., 2020), lipid metabolism, and angiogenesis (Barwari et al., 2016; Du et al., 2021). By affecting microRNA-mRNA networks, astaxanthin can alter signaling pathways and cell death in cardiovascular disease (Chaboksafar et al., 2022), type 2 diabetes (Shokri-Mashhadi et al., 2021), Parkinson’s disease (Shen et al., 2021), and tumors (Faraone et al., 2020). These effects suggest that astaxanthin-induced expression of microRNAs can be used to assess therapeutic effects. However, it is not known whether the protective effects of astaxanthin in myocardial A/R are related to microRNA expression.

Rat cardiomyocyte H9c2 cells are immortalized cells with a cardiac phenotype; H9c2 cells are the most commonly used cell type for isolated primary cardiomyocyte characteristics, they are easily accessible and cultured (Chou et al., 2010; Kuznetsov et al., 2015; Upadhyay et al., 2020; Hu et al., 2022), and they have been used as models in ischemia and reperfusion studies (Han et al., 2004; Wang et al., 2016; Shengm et al., 2019; Luo et al., 2020; Ma et al., 2020). Thus, we analyzed the expression of microRNAs following astaxanthin treatment in an anoxia-reoxygenation system for H9c2 cardiomyocytes to uncover targets of astaxanthin action and guide strategies for myocardial A/R protection.

## 2 Materials and methods

### 2.1 Reagent preparation

Astaxanthin (purity ≥98%, A114383, Aladdin), and Trolox (T137260-1g, Aladdin) were dissolved in DMSO, ensuring that the DMSO content in the cell culture medium was less than 1%. Anoxic solution was composed of NaH_2_PO_4_ 0.9 mM, NaHCO_3_ 6 mM, CaCl_2_ 1.8 mM, MgSO_4_ 1.2 mM, sodium lactate 40 mM, HEPES 20 mM, NaCl 98.5 mM, and KCl 10 mM, pH 6.8. Reoxygenation solution (Chen et al., 2020) was NaCl 129.5 mM, KCl 5 mM NaH_2_PO_4_ 0.9 mM, NaHCO_3_ 20 mM, CaCl_2_ 1.8 mM, MgSO_4_ 1.2 mM, glucose 5.5 mM, and HEPES 20 mM, pH 7.4.

### 2.2 Cell culture and an anoxia-reoxygenation model *in vitro*

**FIGURE 1:**
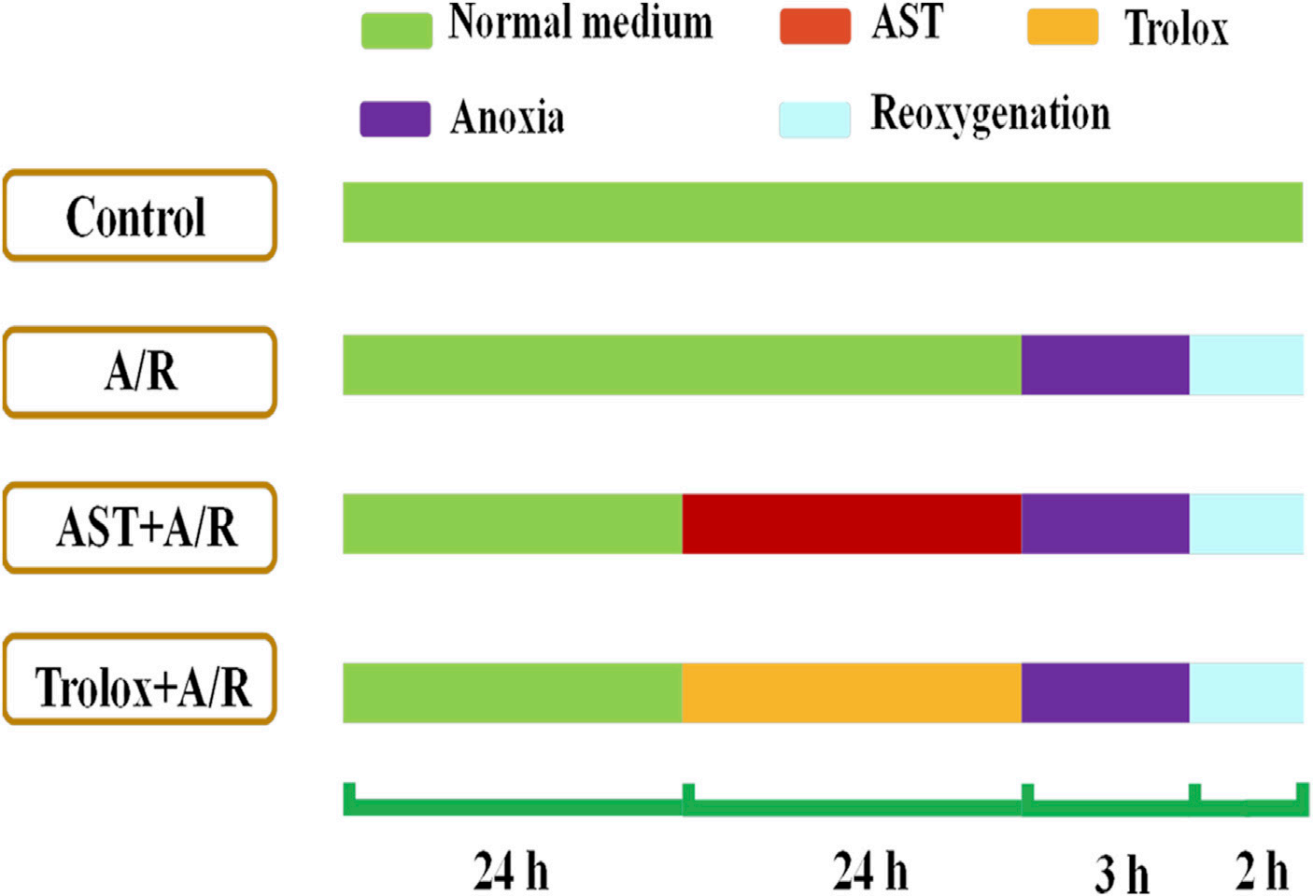
Astaxanthin pretreatment scheme. Cardiomyocytes were pretreated with astaxanthin (AST, 0.313 –5 μM) for 24 h before A/R injury.

H9c2 cells (GNR-5, Stem Cell Bank, Chinese Academy of Sciences, Shanghai, China) were cultured in DMEM (11995073, Gibco, United States) with 10% (v/v) fetal bovine serum (F8192, Sigma) and 1% (v/v) penicillin-streptomycin solution (Gibco) at 37°C with 5% CO_2_ and 95% air. Cells were pretreated for 24 h with astaxanthin (0.312, 0.625, 1.25, 2.5, and 5 μM); Trolox (1.25 μM) was used as a positive control. To simulate myocardial ischemia-reperfusion *in vitro*, a cardiomyocyte anoxia-reoxygenation model was constructed. The anoxic solution was pre-warmed in a 37°C water bath for 30 min and ventilated with a mixture of 95% N_2_ and 5% CO_2_ for 50 min. The cells were washed twice with pre-warmed saturated anoxic solution, an appropriate amount of pre-saturated anoxic solution was added, the cells were placed in an anoxic device and 95% N_2_-5% CO_2_ was introduced for 30 min. Then the device was clamped shut and incubated for 2.5 h in a 37°C incubator (without gas introduction). The reoxygenation solution was similarly pre-warmed and passed through a mixture of 95% O_2_-5% CO_2_ for 50 min. After establishing acute anoxia, the cells were washed twice with the reoxygenation solution, an appropriate amount of pre-saturated reoxygenation solution was added, the cells were placed in a reoxygenation device and 95% O_2_-5% CO_2_ was introduced for 30 min. Then the device was clamped shut and placed in a 37°C incubator (without gas introduction) for 1.5 h before indicators were measured, show as Figure 1.

### 2.3 Cell viability and lactate dehydrogenase assay

Cell viability assays were performed using the CCK-8 kit (BS350A, Biosharp) on 96-well plates according to the manufacturer’s instructions. CCK-8 reagent (10 μL) was added to each well and incubated for 1–2 h at 37°C in the dark. The absorbance was measured at 450 nm using an enzyme marker (PerkinElmer Victor 1420, United States).

Cultures from each treatment subgroup were collected and the lactate dehydrogenase (LDH) content in the supernatant was measured (kit A020-2-2, built in Nanjing, China) to assess cell damage according to the kit instructions.

### 2.4 Antioxidant capacity

Antioxidant enzymes are crucial for cells to dispel oxygen radicals and maintain redox homeostasis. Cells from each treatment group were homogenized; protein content was measured by BCA. Superoxide dismutase (SOD), glutathione peroxidase (GSH-Px), and malondialdehyde (MDA) kits (A001-3-2, A005-1-2, A003-4-1 built in Nanjing, China) were used to assess whether astaxanthin upregulated the antioxidant capacity of H9c2 cardiomyocytes after A/R injury.

### 2.5 Immunoblotting

Cells were collected after 24 h of astaxanthin pretreatment and anoxic reoxygenation. Cells were lysed for 20 min in RIPA buffer (HYK1001, MedChem Express, NJ, United States) containing protease inhibitors and then centrifuged for 15 min at 4°C, 5,550 ×*g*. A BCA kit was used to measure the protein content of the supernatants. The primary antibodies used in the experiment were specific for nuclear factor erythroid-2-related factor 2 (Nrf2), heme oxygenase 1 (HO-1), Bax, Bcl-2, caspase-3, caspase-8, GAPDH, and LaminB. The secondary antibodies were anti-rabbit immunoglobulin and anti-mouse immunoglobulin.

### 2.6 RNA extraction

The cells were diluted with 1×10^5^/DMEM and then seeded on 10 cm diameter plates, pretreated with astaxanthin for 24 h, and collected after anoxic-reoxygenation. Total RNA was extracted. Nanophotometer spectrophotometer was used for assessment of RNA purity (OD260/280 and OD260/230 ratio).

### 2.7 High-throughput RNA sequencing

We used high-throughput sequencing to measure the abundance of microRNAs of the control, A/R, and A/R+astaxanthin groups. The experiment was performed at The Beijing Genomics Institute. Differentially expressed microRNAs were defined by log2 values, |log2 change values| ≥ 0.585, *p* < 0.05, or *q* < 0.01.

### 2.8 Reverse transcription-quantitative polymerase chain reaction analysis

Extracted RNA was converted to cDNA with reverse transcriptase. The primers were selected for the tailing method and real-time PCR was performed using the SYBR-Green polymerase chain reaction kit (Shanghai Bioengineering Technology Co., Ltd.). On the LightCycler^®^ 96 Real-Time Polymerase Chain Reaction System, operations were conducted according to the manufacturer’s instructions. Table 1 contains the PCR primer sequences. The expression levels of microRNAs were normalized to U6 snRNA. Statistical treatment was performed using a t-test with a threshold of *p* < 0.05.

**TABLE 1:** RT-qPCR primer sequences.

Gene		Forward primers
novel-rno-miR86-3p		GTGCTGACCCCTGCGAT
rno-let-7c-1-3p		AGC​TGT​ACA​ACC​TTC​TAG​CTT​TCC
rno-miR-125b-2-3p		ACA​AGT​CAG​GCT​CTT​GGG​A
rno-miR-451-5p		AAC​ACG​TGA​AAC​CGT​TAC​CAT​T
rno-miR-125b-5p		TCC​CTG​AGA​CCC​TAA​CTT​GTG
rno-miR-146a-5p		CCT​GAG​AAC​TGA​ATT​CCA​TGG​GTT
U6		GCT​TCG​GCA​GCA​CAT​ATA​CTA​AAA

### 2.9 KEGG and GO analysis

The KEGG and GO databases were used to identify the functions of microRNAs and the signaling pathways in which they are involved, and to assess the microRNA-mRNA regulatory interactions in astaxanthin-treated H9c2 cardiomyocytes.

### 2.10 Statistical analysis

All experiments were repeated three times and statistical analysis was performed using Graphpad 8.0, SPSS statistical software. *p*-values were analyzed using one-way ANOVA and Tukey’s post-hoc test; *p* < 0.05 was considered statistically significant.

## 3 Results

### 3.1 Effect of astaxanthin pretreatment on survival and lactate dehydrogenase content of anoxia reoxygenation-injured H9c2 cells

**FIGURE 2:**
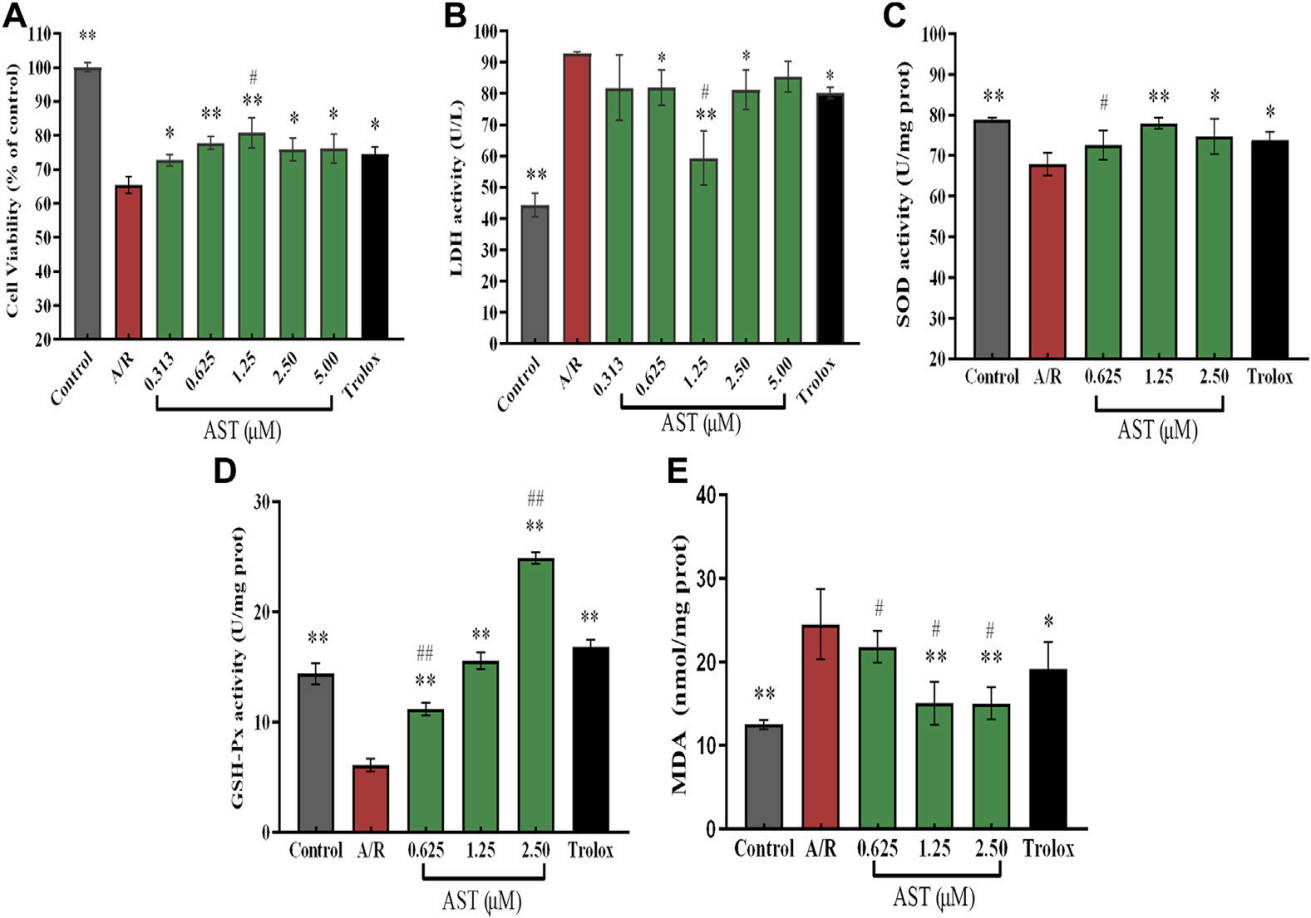
Astaxanthin alleviates A/R damage to H9c2 cardiomyocytes by maintaining cellular activity **(A)**, increasing expression of antioxidant enzymes **(C,D)** and reducing LDH and MDA levels **(B, E)**. *, vs. A/R group (*p < 0.05; **p < 0.01); ^#^, astaxanthin vs. Trolox group (^#^ *p* < 0.05; ^##^ *p* < 0.01). A/R, anoxia reoxygenation; AST, astaxanthin.

We found that the cardiomyocyte survival of the A/R group was 65%, which was significantly reduced compared with the control group. The lactate dehydrogenase (LDH) content in the cell supernatant was about 95 U/L, and twice as high as that of the control group (*p* < 0.01; Figures 2A,B); these results were similar to findings of Liu et al. (2021). Compared with the A/R group, the astaxanthin group showed a dose-dependent increase in cell survival and decreasing supernatant lactate dehydrogenase level (*p* < 0.05 and *p* < 0.01). In addition, the Yaghooti et al. found that astaxanthin significantly improved cell survival of mesenchymal stem cells after palmitate injury (Yaghooti et al., 2019). On the basis of these results, we selected astaxanthin concentrations of 0.625, 1.25, and 2.5 μM for subsequent experiments.

### 3.2 Effect of astaxanthin pretreatment on superoxide dismutase, glutathione peroxidase, and malondialdehyde levels in H9c2 cardiomyocytes after A/R injury

As shown in Figures 2C–E, superoxide dismutase (SOD) and glutathione peroxidase (GSH-Px) activities were significantly decreased by 11.3 U/mgprot and 8 U/mgprot (*p* < 0.01), and the level of malondialdehyde (MDA) was significantly increased by 12 nmol/mgprot (*p* < 0.01) in the A/R group compared with the control group. The activities of SOD and GSH-Px were significantly increased and MDA level was significantly lower in the astaxanthin group compared with the A/R group (*p* < 0.05 or *p* < 0.01). At an astaxanthin concentration of 1.25 μM, the activity of SOD and GSH-Px increased to 78 U/mgprot and 15.2 U/mgprot, which was higher than 11 U/mgprot and 10 U/mgprot in A/R group. The MDA level was significantly reduced to 14.7 nmol/mgprot, approximately 10 nmol/mgprot lower than the A/R group. Compared with the Trolox positive control group, the SOD and GSH-Px activities were slightly lower in the 0.625 μM astaxanthin concentration group (*p* < 0.05 or *p* < 0.01), and the MDA content was slightly higher in the Trolox group (*p* < 0.05). At a concentration of 1.25 μM astaxanthin, compared with the Trolox group, SOD activity was slightly higher, GSH-Px activity was the same, and MDA content was significantly decreased by 6 nmol/mgprot (*p* < 0.05). At an astaxanthin concentration of 2.5 μM, the SOD activity was on par with the Trolox group, GSH-Px activity was significantly increased by 8 U/mgprot (*p* < 0.01), and MDA content was significantly lower (*p* < 0.05) than the Trolox group. Su et al. (2022) studied the trend of various antioxidant enzymes in kidney I/R injury. As a powerful natural antioxidant, astaxanthin protects against damage and maintains redox balance by activating antioxidant enzymes. Astaxanthin protects against hypertensive vascular remodeling injury by increasing the level of the SOD that prevents oxidative injury (Chen et al., 2020).

### 3.3 Effect of astaxanthin pretreatment on Bcl-2, Bax, and capase-8/caspase-3 in H9c2 A/R-treated cardiomyocytes

We found that Bcl-2/Bax ratio were reduced and expression of caspase-8 and caspase-3 increased in each group with A/R injury compared with the normal group (*p* < 0.01; Figures 4A–C). Bcl-2/Bax ratio were significantly higher in the astaxanthin groups compared with the A/R group, and the expression of caspase-3 and caspase-8 was also significantly lower (*p* < 0.05 or *p* < 0.01). Bcl-2/Bax ratio were higher in the astaxanthin group compared to the Trolox positive control group (*p* < 0.01). Many studies have shown that astaxanthin can inhibit apoptosis (Cui et al., 2020; Zhang et al., 2021; Abbaszadeh et al., 2022).

### 3.4 Effect of astaxanthin pretreatment on nuclear Nrf2/HO-1 pathway in H9c2 cardiomyocytes after A/R injury

**FIGURE 3:**
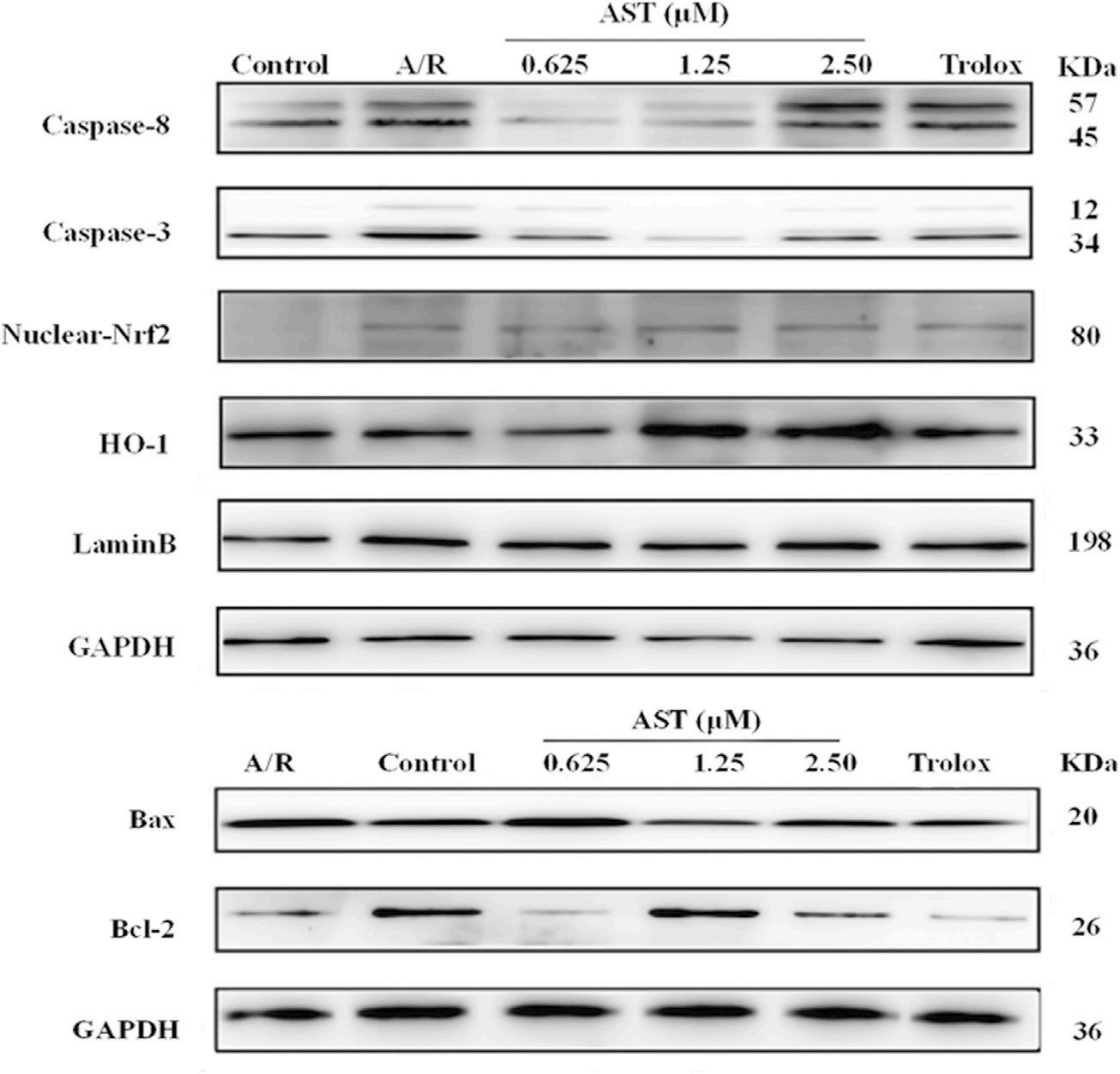
Immunoblots of protein content. A/R, anoxia reoxygenation; AST, astaxanthin.

**FIGURE 4:**
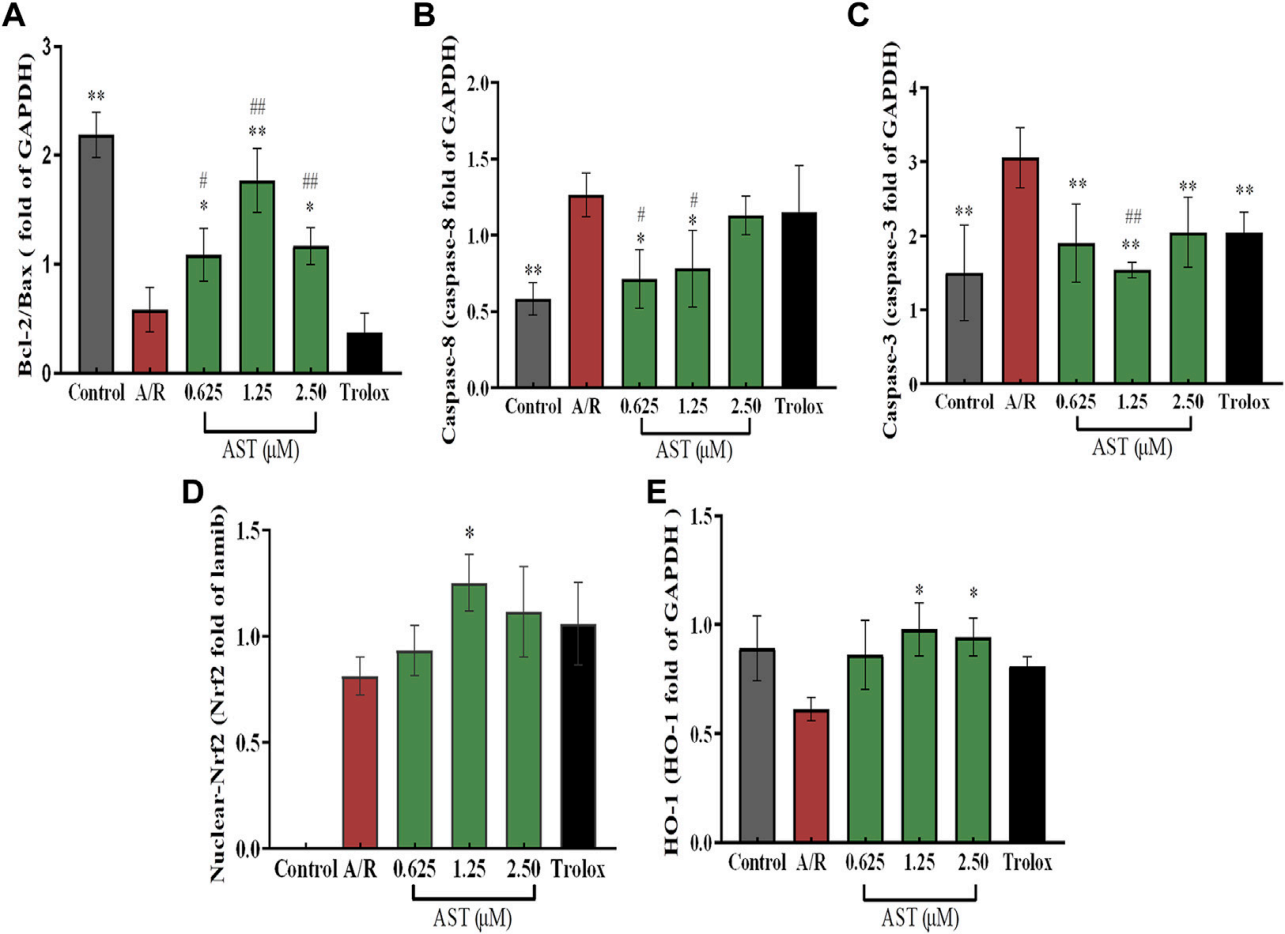
Astaxanthin pretreatment reduced apoptosis-related protein content **(A–C)** and enhanced nuclear Nrf2 and HO-1 protein content **(D,E)** in cardiomyocytes after A/R injury. *, vs. A/R group (*p < 0.05; **p < 0.01); ^#^, astaxanthin vs. Trolox group (^#^ *p* < 0.05; ^##^ *p* < 0.01). A/R, anoxia reoxygenation; AST, astaxanthin.

The Nrf2/HO-1 pathway is an important antioxidant mechanism. We investigated the effect of astaxanthin treatment on the Nrf2/HO-1 pathway in A/R-injured H9c2 cardiomyocytes (Figure 3). Oxidative stress caused by A/R injury increased Nrf2 nuclear translocation and decreased HO-1 content (*p* < 0.05). Astaxanthin increased the nuclear translocation of Nrf2 and the amount of HO-1 compared with the A/R group (*p* < 0.05 and *p* < 0.01, Figures 4D, E). In addition, the expression level of HO-1 protein in the control group was higher than that in the A/R group. This finding suggested that the function of cells and mitochondria would be affected, and cell homeostasis would be unbalanced due to the abnormal production of reactive oxygen species and energy metabolism during anoxia-reoxygenation injury, thus resulting in the decrease of HO-1 protein synthesis. Second, inhibitors of Nrf2, such as Bach1, also inhibit the synthesis of HO-1 (Lignitto et al., 2019); Researchers indicated that inhibition of PI3K-Akt pathway can significantly reduce the expression of HO-1 protein, thus weakening the protective effect of HO-1 on cardiomyocytes (Sun et al., 2012). Astaxanthin alleviates anoxia-reoxygenation injury in cardiomyocytes and maintains cellular vitality. Therefore, in the presence of astaxanthin, the increase of Nrf2 can promote an increase of HO-1.

### 3.5 Regulatory network of microRNAs for astaxanthin pretreatment protection against A/R injury in H9c2 cardiomyocytes

#### 3.5.1 Analysis of RNA sequencing data

To investigate a link between the protective effect of astaxanthin against A/R damage of cardiomyocytes and microRNA regulation, we constructed a control group, an A/R group, and an astaxanthin+A/R group. After filtering for poor-quality tags, splice contaminants, and sequences with less than 18 nucleotides, >90.8% clean tags were obtained from each group (Supplementary Table SA1; Supplementary Datasheet S1). These tags were between 20–24 nt, with a peak at 23 nt (Supplementary Figures SA1–SA3), and they were comparable in size to animal microRNAs. Taking AR2 as an example, based on the miRBase database, the intergenic, mature sequences, and unmap tags accounted for 28.57%, 22.34%, and 15.44%, respectively (Table 2).

**TABLE 2:** Summary of small RNA classification (AR2).

Class	Count	Percent (%)
Total	14,060,479	100.00
intergenic	4,016,970	28.57
Mature	3,141,180	22.34
Rfam other sncRNA	358,546	2.55
SnRNA	2,364	0.02
Unmap	2,170,699	15.44
Intron	357	0.00
RRNA	386,539	2.75
Hairpin	2,547,279	18.12
SnoRNA	46,345	0.33
Precursor	1,233,307	8.77
Exon	167	0.00
Repeat	95,171	0.68
TRNA	61,555	0.44

#### 3.5.2 Screening for differentially expressed microRNAs

**FIGURE 5:**
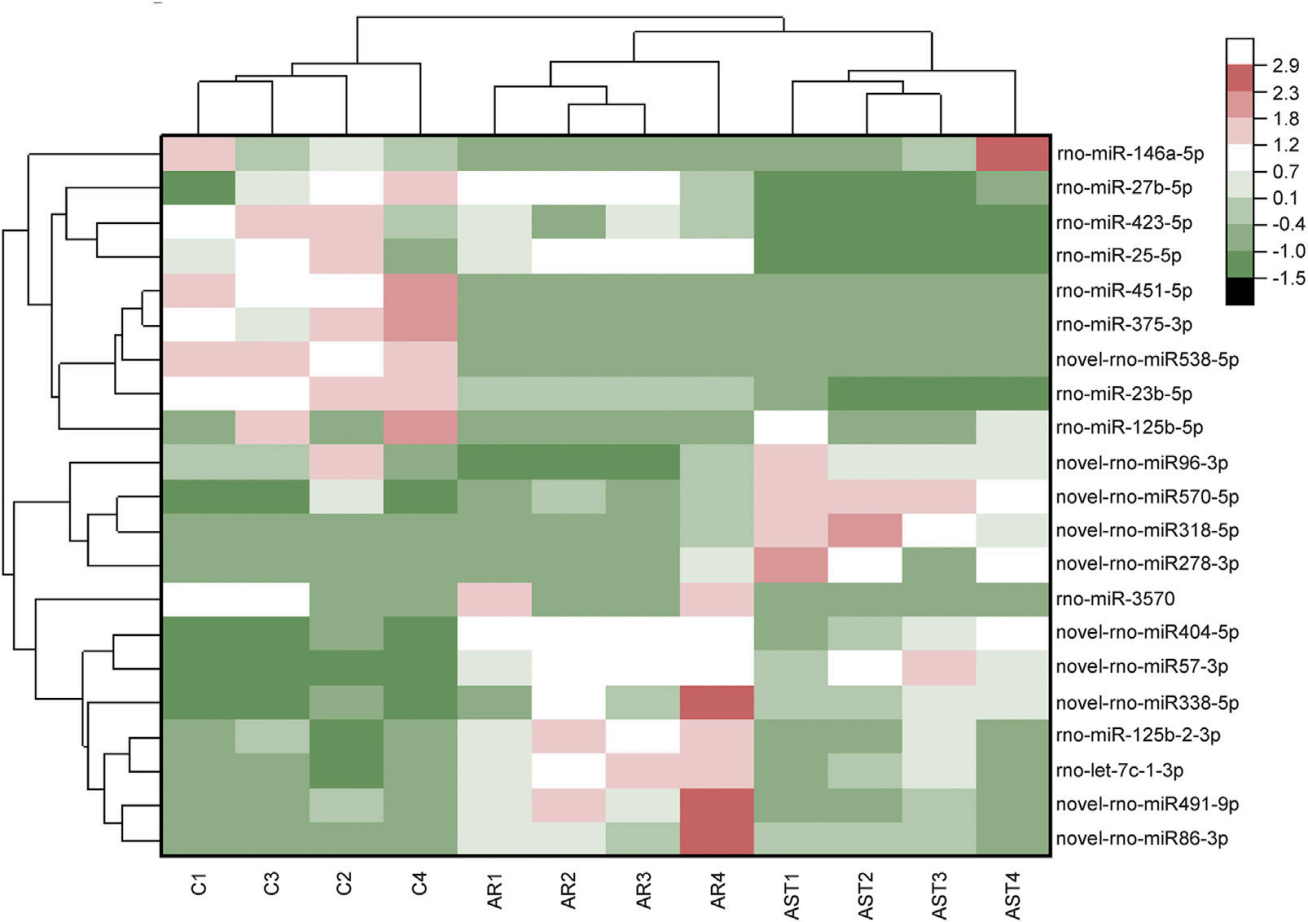
RNA-Seq-based differential microRNA heat maps were plotted against the expression level of each sample.

The screening criteria for differentially expressed microRNAs were *q* < 0.05 and |log2 change value| ≥ 0.585. Tables 3, 4 and Figure 5 show that the number of differential microRNAs identified in the AR/C group was 47 (14 upregulated and 33 downregulated) and the number of differential microRNAs in the AR/astaxanthin group was 16 (7 upregulated and 9 downregulated). To identify the key microRNAs whereby astaxanthin exerted a protective effect and selecting microRNAs that astaxanthin was capable of back-regulating abnormal changes in AR, three eligible microRNAs were found, of which rno-miR-125b-5p, rno-miR-146a-5p were upregulated and rno-let-7c-1-3p was downregulated.

**TABLE 3:** Data related to differentially expressed microRNAs in the AR/C group.

Name	log2	Q-value	Style
novel-rno-miR338-5p	2.5049633545	0.0013447813	Up
novel-rno-miR404-5p	2.1303897332	0.0000000000	Up
novel-rno-miR491-3p	1.7834253004	0.0007953169	Up
novel-rno-miR57-3p	2.6927065408	0.0000000000	Up
novel-rno-miR86-3p	5.1348697435	0.0000034224	Up
rno-let-7c-1-3p	1.3034415883	0.0000000000	Up
rno-miR-125b-2-3p	0.7021274519	0.0000001958	Up
rno-miR-1306-5p	0.5932718160	0.0033475039	Up
rno-miR-145-5p	0.6932031287	0.0005473485	Up
rno-miR-15b-3p	0.6163100658	0.0002558517	Up
rno-miR-1839-3p	1.4202202386	0.0014064690	Up
rno-miR-192-3p	0.8226268106	0.0045304967	Up
rno-miR-194-3p	2.0023003993	0.0170973573	Up
rno-miR-25-3p	0.6613644772	0.0000073839	Up
novel-rno-miR202-5p	−2.7128867320	0.0135343649	Down
novel-rno-miR230-5p	−1.4379035794	0.0000000000	Down
novel-rno-miR395-3p	−1.5487219681	0.0123236801	Down
novel-rno-miR41-3p	−0.7906107345	0.0258212827	Down
novel-rno-miR412-3p	−1.0145987798	0.0032398711	Down
novel-rno-miR538-5p	−3.7888119604	0.0000000000	Down
novel-rno-miR596-5p	−0.6990842271	0.0008643809	Down
rno-miR-125b-5p	−6.6007959687	0.0002191810	Down
rno-miR-127-3p	−5.0452663118	0.0312976807	Down
rno-miR-128-1-5p	−1.5110878673	0.0000000002	Down
rno-miR-135b-5p	−6.3086119741	0.0000000000	Down
rno-miR-141-3p	−2.6543705717	0.0000000630	Down
rno-miR-144-3p	−4.4983598054	0.0000000041	Down
rno-miR-146a-5p	−4.5154835074	0.0000000000	Down
rno-miR-150-5p	−1.0573660920	0.0016429998	Down
rno-miR-191a-3p	−0.9717242231	0.0000000000	Down
rno-miR-195-3p	−2.0145167679	0.0000000044	Down
rno-miR-200a-3p	−3.8920011457	0.0000008808	Down
rno-miR-200b-3p	−1.9048423741	0.0012647313	Down
rno-miR-200c-3p	−2.6972178196	0.0000008743	Down
rno-miR-219a-1-3p	−0.7941127455	0.0079400779	Down
rno-miR-223-3p	−5.1140115873	0.0006508249	Down
rno-miR-23b-5p	−0.7947085467	0.0003646666	Down
rno-miR-30c-1-3p	−1.1761770757	0.0000000001	Down
rno-miR-340-5p	−5.7381831631	0.0000882814	Down
rno-miR-351-3p	−1.2436001787	0.0000000000	Down
rno-miR-374-3p	−0.8972095204	0.0000238045	Down
rno-miR-375-3p	−5.8839686756	0.0000000000	Down
rno-miR-451-5p	−7.9158299205	0.0000000000	Down
rno-miR-615	−2.1171326869	0.0019310161	Down
rno-miR-702-5p	−2.2289173918	0.0000000000	Down
rno-miR-760-3p	−0.7426714161	0.0016162673	Down
rno-miR-96-5p	−1.6586398439	0.0000647403	Down

**TABLE 4:** Data related to differentially expressed microRNAs in the AR/astaxanthin group.

Name	log2	Q-value	Style
novel-rno-miR191-5p	3.0215221471	0.0387427706	Up
novel-rno-miR3-3p	1.7947981113	0.0391981639	Up
novel-rno-miR318-5p	3.6153831264	0.0000032156	Up
novel-rno-miR570-5p	2.0826155275	0.0000000016	Up
novel-rno-miR96-3p	1.1786310058	0.0009818513	Up
rno-miR-125b-5p	6.1346748243	0.0016613904	Up
rno-miR-146a-5p	4.5387486773	0.0339128086	Up
rno-let-7c-1-3p	−0.6400168626	0.0128194050	Down
rno-miR-23a-5p	−1.2171899250	0.0154065232	Down
rno-miR-23b-5p	−0.9468575897	0.0000041168	Down
rno-miR-25-5p	−0.9486004837	0.0000000000	Down
rno-miR-27a-5p	−0.8386286942	0.0011937948	Down
rno-miR-27b-5p	−0.7869440573	0.0128194050	Down
rno-miR-351-3p	−0.7814778593	0.0231874503	Down
rno-miR-3570	−7.7863261987	0.0128194050	Down
rno-miR-423-5p	−0.9770008881	0.0000032156	Down

**FIGURE 6:**
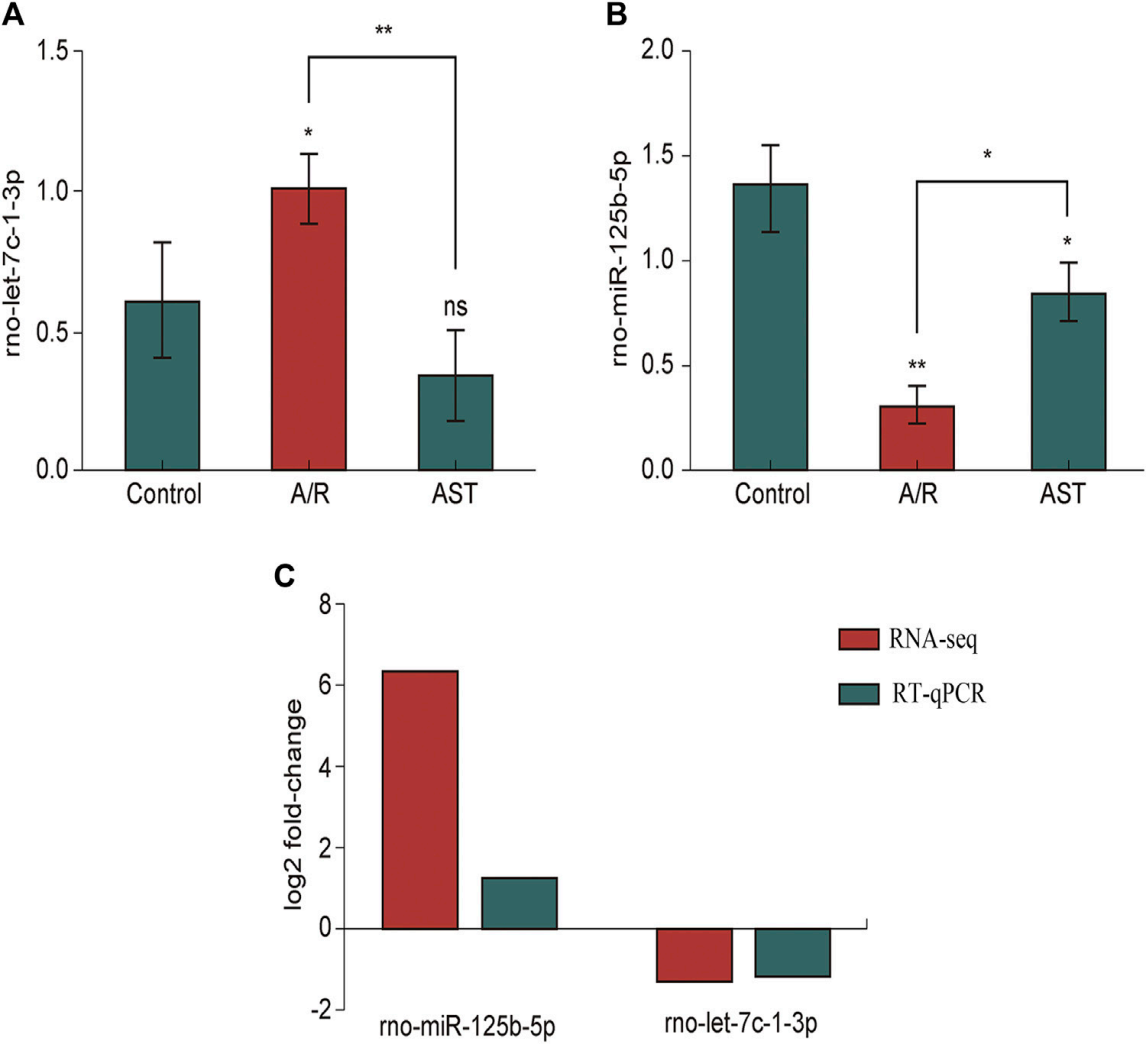
RT-qPCR analysis of the expression of rno-miR125b-5p **(A)**, Let-7c-1-3p **(B)**. **(C)** Comparison of RNA-seq and RT-qPCR results for selected differentially expressed microRNAs (*p < 0.05; **p < 0.01). A/R, anoxia reoxygenation; AST, astaxanthin.

As verified by qPCR (e.g., Figures 6A–C), we found that the expression of rno-miR-125b-5p and rno-let-7c-1-3p in each treatment group was consistent with the sequencing results, and the qPCR results were generally in good agreement with the RNA-seq results.

#### 3.5.3 Prediction of microRNA target genes and their enrichment

We used the TargetScan (8.0), microRNAda (v3.3a), and RNAhybrid databases to predict target genes for rno-miR-125b-5p and rno-let-7c-1-3p.

**FIGURE 7:**
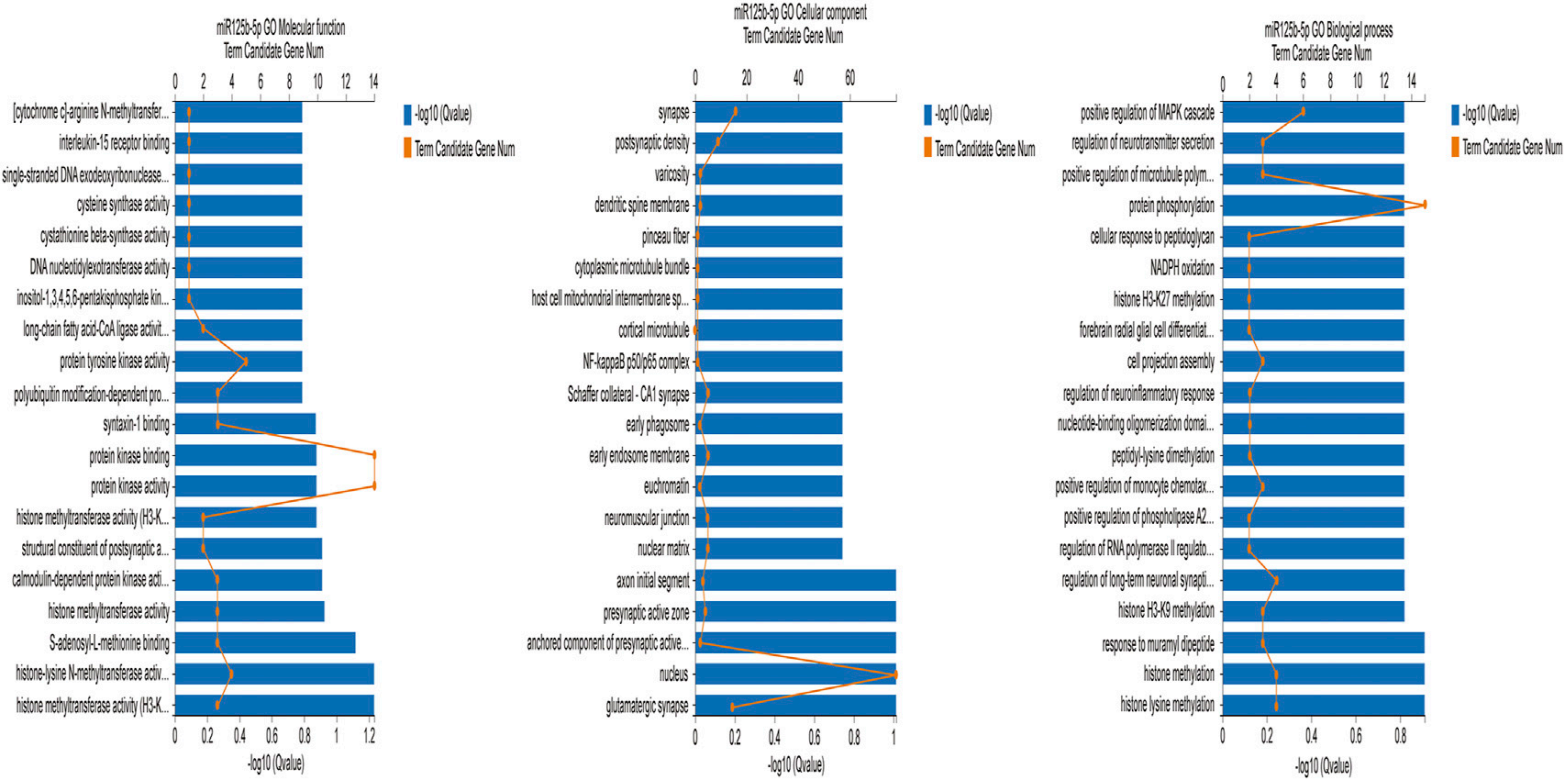
GO functional classification of differentially expressed rno-miR125b-5p target genes.

We used the GO database (http://www.geneontology.org) to identify enrichment of the target genes. The GO database is a comprehensive overview of the gene functions of an organism divided into three ontologies, namely Biological process, Cellular component, and Molecular function (Wang et al., 2021). Because rno-let-7c-1-3p had no more than ten target genes, we analyzed rno-miR-125b-5p. Figure 7 is the GO enrichment analysis of rno-miR-125b-5p target genes (*q* ≤ 0.01). The target genes were associated with branching involved in biological processes such as histone lysine methylation, histone methylation, and protein phosphorylation. The GO cellular components were the nucleus and the synaptic precursor, and the GO molecular functions were histone methyltransferase activity (H3-K9 specific), histone-lysine N-methyltransferase activity, and protein kinase activity.

**FIGURE 8:**
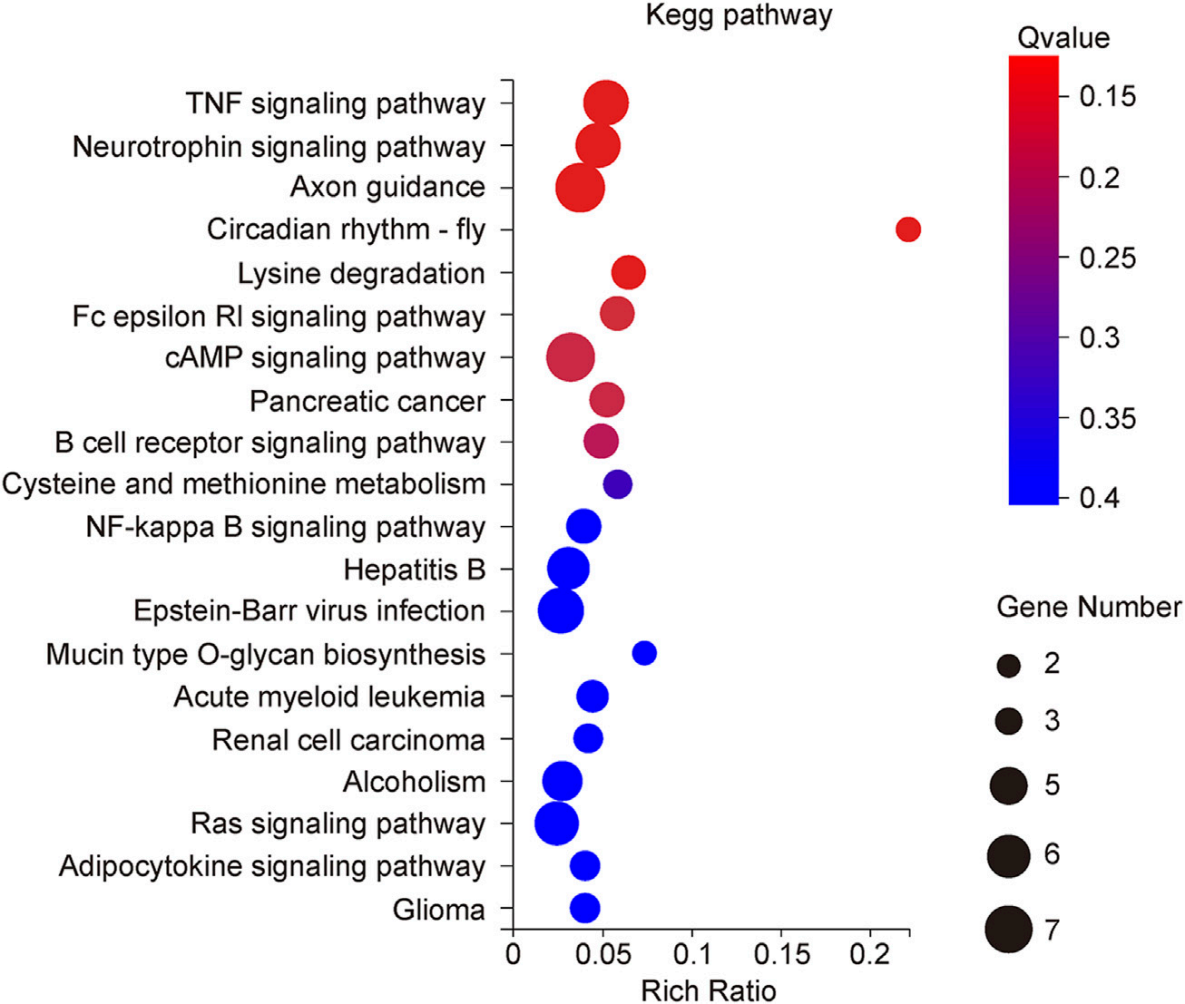
KEGG classification of rno-miR125b-5p target genes.

**FIGURE 9:**
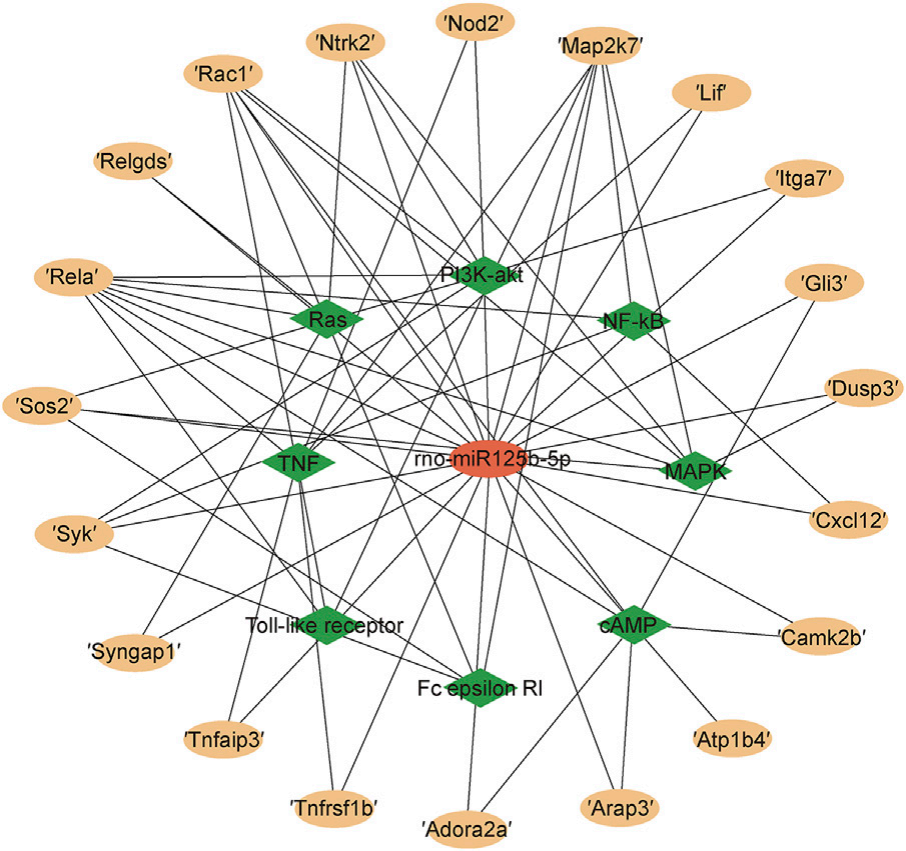
Target mRNAs for rno-miR125b-5p in signaling pathways.

The KEGG Pathway enrichment analysis (Figure 8) showed that the target genes of the differentially expressed microRNAs across treatments were significantly enriched in the TNF signaling pathway, axon guidance, and the NF-κB pathway. The target genes of the TNF signaling pathway enriched by miR125b-5p were Tnfrsf1b, Nod2, Rela, Map2k7, Lif, and Tnfaip3; the NF-κB signaling pathway contains target genes that Rela, Map2k7, Syk, and Cxcl12. The Ras signaling pathway contains the target genes Syngap1, Ntrk2, Ralgds, Rela, Rac1, and Sos2; the MAPK signaling pathway contains the target genes Ntrk2, Rela, Map2k7, Rac1, Dusp3, and Sos2; the PI3K-AKT signaling pathway contains the target genes Ntrk2, Syk, Rela, Rac1, Itga7 and Sos2; the FC epsilon RI signaling pathway contains the target genes Syk, Map2k7, Rac1, and Sos2; the Toll-like receptor signaling pathway contains the target genes Rela, Map2k7, Rac1 (Figure 9). Rela, Rac1, and Map2k7 target genes participate in multiple signaling pathways and may be associated with protection against myocardial anoxia-reoxygenation damage; thus, these genes are value for follow-up studies.

## 4 Discussion

Astaxanthin efficiently scavenges superoxide anions, hydroxyl radicals, and lipid-free radicals (Zuluaga et al., 2018). Many studies on the cardioprotective effects of astaxanthin demonstrate its powerful antioxidant activity in prevention and treatment of ischemia-reperfusion lesions *in vivo* (Xue et al., 2019; Wang et al., 2021). And astaxanthin has been reported to protect the myocardium from ischemia/reperfusion injury by decreasing oxidative stress and apoptosis (Adluri et al., 2013). Moreover, the beneficial effects of astaxanthin on IRI-induced increase in plasma and cardiac levels of oxidative stress (Pongkan et al., 2017). Similar effects have been observed in an *in vitro* study, wherein administration of astaxanthin protected myocardial cells from anoxia/reoxygenation injury (Gai et al., 2020). However, the mechanism of astaxanthin protection against myocardial ischemia-reperfusion injury was unknown. We studied the mechanism of myocardial ischemia-reperfusion injury *in vitro*.

When myocardium undergoes ischemia-reperfusion injury, myocardial cell permeability increases, and a large amount of lactate dehydrogenase (LDH) is released; thus, extracellular LDH activity can reflect the degree of myocardial cell damage (Gai et al., 2020). Our LDH measurements showed that, compared to the anoxia reoxygenation group, astaxanthin improved the survival of H9c2 cardiomyocytes that would have had significant A/R injury and LDH leakage (Figures 2A,B). Excess reactive oxygen species is a major contributor to myocardial ischemia-reperfusion injury because it leads to oxidative stress that, in turn, causes apoptosis of cardiomyocytes. Antioxidant enzyme activity decreases when cells are damaged. SOD can remove superoxide anions to produce H_2_O_2_. GSH-Px catalyzes the conversion of lipid hydroperoxide (LOOH) produced during lipid peroxidation to the corresponding alcohol lipid hydroxide (LOH), blocking the side chain cycle reaction of lipid peroxidation. We found that SOD and GSH-Px levels were reduced and MDA level was increased in the A/R group compared with the normal group. In the astaxanthin group, intracellular SOD and GSH-Px levels were increased, and malondialdehyde level was decreased in cardiac myocytes (Figures 2C–E). The results suggested that astaxanthin can scavenge free radicals, increase the activity of antioxidant enzymes, and reduce oxidative damage during A/R.

Anoxia reoxygenation promotes cardiomyocyte expression of apoptosis-related proteins such as caspase-8 and caspase-3, initiates apoptotic signaling pathways, and induces apoptosis in cardiomyocytes. The astaxanthin group had significantly reduced A/R damage in cardiomyocytes and increased expression of Bcl-2, activated caspase-8/caspase-3, and delayed onset of apoptosis (Figures 4A–C). All these results confirmed that astaxanthin can alleviate oxidative damage during myocardial ischemia-reperfusion.

Heme oxygenase-1 (HO-1) is involved in preventing oxidative stress and in anti-apoptotic processes; HO-1 has an important protective function in A/R injury. Nuclear factor erythroid 2-related factor 2 (Nrf2) is present in oxygen-depleted organs and is an important regulator of oxidative stress (Cui et al., 2020). The Nrf2/HO-1 signaling pathway is an important intracellular antioxidant pathway. In response to oxidative stress, Nrf2 dissociates from kelch-like ECH-associated protein 1 and translocates from the cytoplasm to the nucleus to induce antioxidant and detoxification genes (Kohandel et al., 2021). Astaxanthin activated the Nrf2/HO-1 pathway to suppress oxidative stress, alleviating myocardial injury and ischemia-reperfusion injury of femur and liver. Astaxanthin alleviates cardiomyocyte apoptosis after coronary microembolization by inhibiting oxidative stress *via* Nrf2/HO-1 pathway in rats (Zuluaga et al., 2019; Cui et al., 2020). We found that the Nrf2/HO-1 pathway was adaptively activated to counteract A/R damage of cardiomyocytes. Astaxanthin pretreatment enhanced the activation of the Nrf2/HO-1 pathway, which promoted the antioxidant stress response (Figures 4D,E). Although there have been numerous studies on the protective effects of astaxanthin in a variety of diseases, to the best of our knowledge, our study is the first to demonstrate the contribution of the Nrf2/HO-1 pathway to the protective effects of astaxanthin in myocardial ischemia-reperfusion.

MicroRNAs are a class of small, non-coding RNAs that regulate more than 30% of genes by inhibiting the translation of their mRNAs or promoting mRNA degradation (Poller et al., 2018). Recent studies have identified novel mechanisms of regulatory networks between microRNAs and mRNAs that control the pathophysiology of cardiovascular disease (Gong et al., 2020; Liu et al., 2021). Astaxanthin can protect cardiomyocytes from anoxia/reoxygenation injury by regulating the miR-138/HIF-1α axis (Gai et al., 2020). Therefore, we systematically analyzed the microRNAs that responded to astaxanthin and protected cardiomyocytes from A/R injury.

We used RNA-seq and RT-qPCR validation to identify differentially expressed microRNAs associated with astaxanthin protection against myocardial A/R injury. In our study, rno-miR-125b-5p expression in the three treatment groups was control group >astaxanthin+A/R group >A/R group. Therefore, A/R damage decreased rno-miR-125b-5p expression. MiR-125b-5p has low abundance in patients with acute ischemic stroke and ischemic cardiomyopathy, and miR-125b-5p can be used as a diagnostic biomarker of heart failure (Tiedt et al., 2017; Bayoumi et al., 2018). We found that predicted target genes of rno-miR-125b-5p were enriched in signaling pathways related to oxidative stress, inflammation, and apoptosis. Many studies have shown that microRNAs can treat diseases associated with oxidative stress (Wang et al., 2019). Rno-miR-125b-5p is closely associated with oxidative stress-induced diseases and protects endothelial cells from hydrogen peroxide-induced oxidative stress (Wei et al., 2017). In our data, the rno-miR-125b-5p target gene Rac1 (small Rac family GTPase 1) can activate MAPK and PI3K-AKT signaling pathways. Because the mechanism of anoxia-reoxygenation-induced myocardial injury is crucial, myocardial oxidative stress will generate a large amount of reactive oxygen species, and astaxanthin is a natural antioxidant. Our results also show that rno-miR-125b-5p was a important target in astaxanthin antioxidant effect on myocardial anoxia-reoxygenation injury, and astaxanthin can increase the expression of rno-miR-125-b-5p after anoxia-reoxygenation injury. Therefore, we speculate that rno-miR-125b-5p is a target for astaxanthin’s involvement in protection from myocardial injury.

In addition, miR-125b-5p acts as a protective agent for ischemic cardiomyocytes by inhibiting the pro-apoptotic genes p53, Bak1, and Klf13 (Bayoumi et al., 2018; Zhu et al., 2018). Apoptosis has multiple pathways, such as oxidative stress, inflammation, and DNA damage. We found that the target genes of rno-miR-125b-5p were significantly enriched in the TNF signaling pathway, in which TNF-α could activate three downstream signaling pathways: caspase protease, JNK signaling pathway, and NF-κB signaling pathway. TNF-α dissociates SODD bound to TNFR1 on the cell membrane, exposing the dead zone of TNFR1, and FADD binds to TNFR1 to activate downstream caspase-8 and caspase-3, leading to apoptosis. Astaxanthin reduced the levels of caspase-8 and caspase-3, which suggested that astaxanthin inhibits apoptosis by enhancing the expression of rno-miR-125b-5p. TNFR1 also binds to TRADD, RIPI, and TRAF2/5, activates the IKK kinase complex, phosphorylates and degrades IKBα, separates it from NF-κB, and activates the inflammatory response. MiR-125b-5p suppresses excessive inflammation in human arthritic chondrocytes by downregulating Rela target genes (Rasheed et al., 2019). LIF, a proinflammatory factor regulated by miR-125b-5p, is a protective factor against acutely stressed myocardium during ischemia-reperfusion (Zouein et al., 2013). Further study is needed to determine whether astaxanthin can inhibit the inflammatory response of rno-miR-125b-5p to anoxia-reoxygenation injury.

Although the regulatory function of rno-miR-125b-5p is not fully understood, current data suggest that upregulation of rno-miR-125b-5p is beneficial for protecting against myocardial anoxia-reoxygenation injury. In a follow-up study, we used qPCR for different treatment groups to verify differential expression of Rac1, the target gene of miR125b-5p. Some studies showed that Rac1 activation can aggravate myocardial injury (Wang et al., 2022). Rac1 can activate ERK/P38MAPK signaling pathway (Zhu et al., 2009). In the future, we will investigate whether astaxanthin can inhibit apoptosis by modulating the miR125b-5p/Rac1/ERK/P38MAPK signaling pathway and protect the heart muscle from anoxia and reoxygenation damage by modulating the miR125b-5p/Rac1/ERK/P38MAPK signaling pathway. In addition, several studies have evaluated the antioxidant effects of orally and intravenously administered astaxanthin in I/R models (Gross and Lockwood, 2004; Dilli et al., 2022). We demonstrated that astaxanthin improved cell survival and inhibited oxidative stress and apoptosis after myocardial anoxic-reoxygenation injury. Therefore, our results can serve as a reference for *in vivo* model studies.

## 5 Conclusion

Astaxanthin pretreatment reduced myocardial injury and increased cardiomyocyte survival, and inhibited apoptosis and oxidative stress damage. We identified differentially expressed microRNAs and related target genes that, with astaxanthin pretreatment, protect cardiomyocytes from A/R injury. These target genes can be used to further investigate their regulatory relationships.

## Data Availability

The datasets presented in this study can be found in online repositories. The NCBI serial number: PRJNA908639. https://www.ncbi.nlm.nih.gov/sra/PRJNA908639.
