# Supplementary material for: Effects of astaxanthin on microRNA expression in a rat cardiomyocyte anoxia-reoxygenation model
Source: Front Pharmacol. 2023 Feb 3;14:1103971. doi: 10.3389/fphar.2023.1103971 (PMC9936191; doi:10.3389/fphar.2023.1103971)
Supplement: Supplementary file 1 [file DataSheet1.PDF]

Appendix Material

1 Appendix Table

Appendix Table 1. Quality statistics of raw reads after filtering of each group

sample	RawTag Tag Count	Low Quality Tag Count	Invalid Adapter Count	PloyA Tag Count	Short Valid Count	Clean Tag	Percentage of Clean Tag(%)
AR1	35232153	138135	1150845	8335	1292754	32642084	92.65
AR2	35232153	157011	1374600	7019	1428735	32264788	91.58
AR3	35232153	150631	902949	10625	756645	33411303	94.83
AR4	35232153	167337	1730984	9839	1502529	31821464	90.32
AST1	35232153	155497	695848	4214	538836	33837758	96.04
AST2	35232153	157840	922218	5922	659467	33486706	95.05
AST3	35232153	160283	1045470	5536	714341	33306523	94.53
AST4	35232153	154283	1179291	6861	1024608	32867110	93.29
C1	35232153	128684	1249783	5200	985592	32862894	93.28
C2	35232153	112465	1300320	3541	1212813	32603014	92.54
C3	35232153	111231	1186254	5891	1031556	32897221	93.37
C4	35232153	132813	1963112	8363	1759752	31368113	89.03

2 Appendix Figures

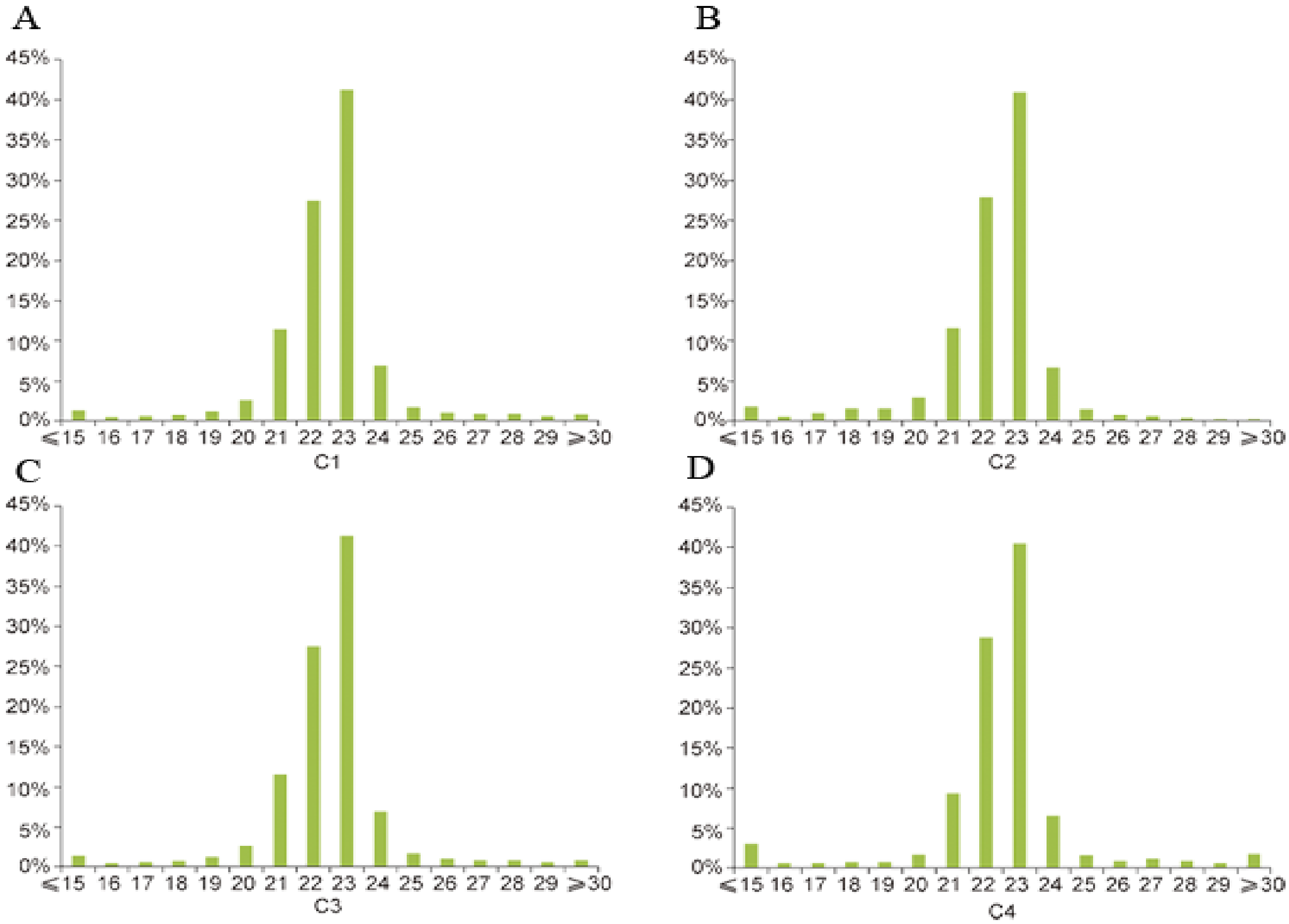

Appendix Figure 1. The distribution of tag length from control group, A-D are four parallel groups.

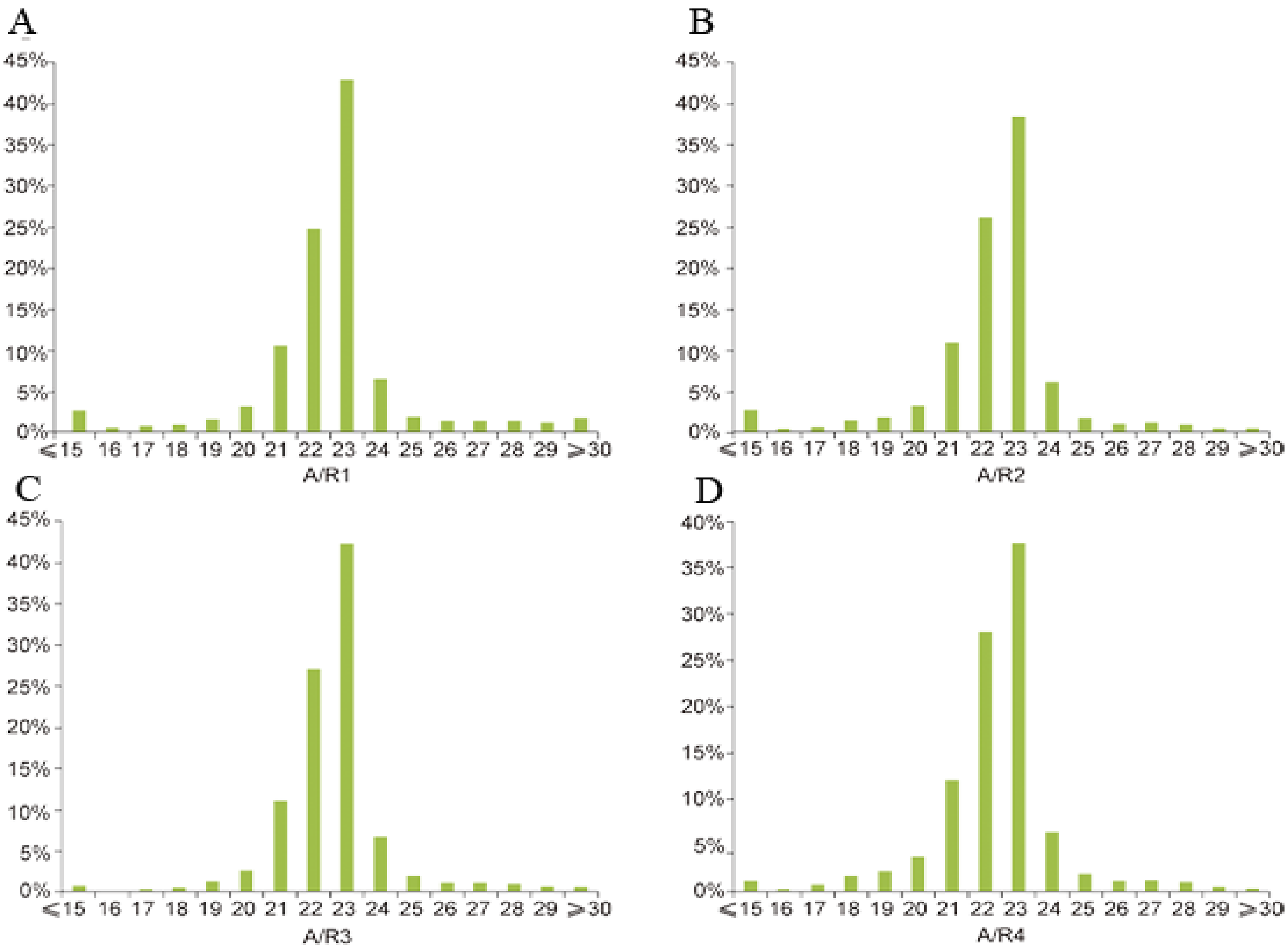

Appendix Figure 2. The distribution of tag length from anoxia reoxygenation group. A-D are four parallel groups.

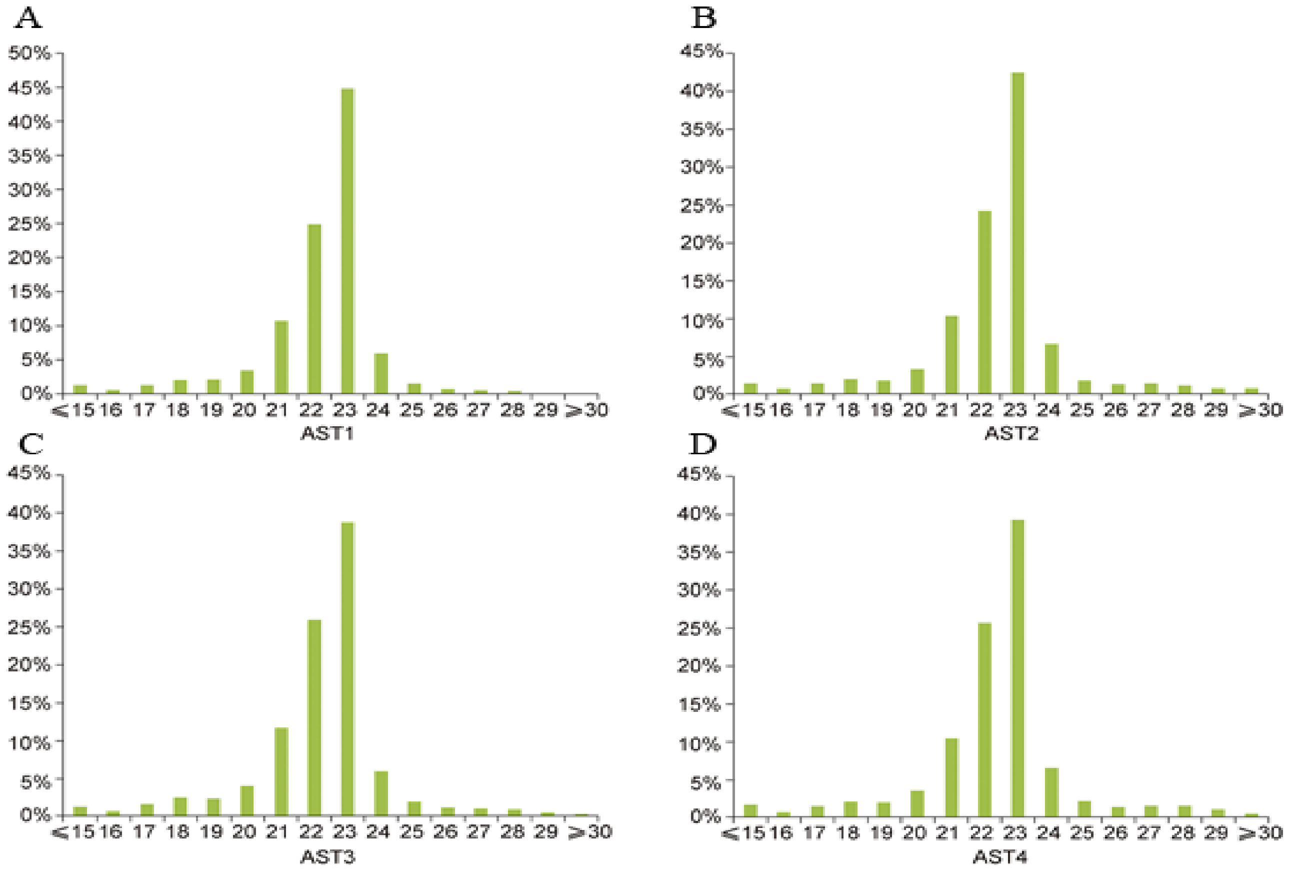

Appendix Figure 3. The distribution of tag length from astaxanthin group. A-D are four parallel groups.
